# Gastroschisis: Antenatal Sonographic Predictors of Adverse Neonatal Outcome

**DOI:** 10.1155/2014/239406

**Published:** 2014-12-22

**Authors:** Rachael Page, Zachary Michael Ferraro, Felipe Moretti, Karen Fung Kee Fung

**Affiliations:** Division of Maternal-Fetal Medicine, The Ottawa Hospital, General Campus, 501 Smyth Road, Room 8472, Ottawa, ON, Canada K1H 8L6

## Abstract

*Objectives*. The aim of this review was to identify clinically significant ultrasound predictors of adverse neonatal outcome in fetal gastroschisis. *Methods*. A quasi-systematic review was conducted in PubMed and Ovid using the key terms “gastroschisis,” “predictors,” “outcome,” and “ultrasound.” *Results*. A total of 18 papers were included. The most common sonographic predictors were intra-abdominal bowel dilatation (IABD), intrauterine growth restriction (IUGR), and bowel dilatation not otherwise specified (NOS). Three ultrasound markers were consistently found to be statistically insignificant with respect to predicting adverse outcome including abdominal circumference, stomach herniation and dilatation, and extra-abdominal bowel dilatation (EABD). *Conclusions*. Gastroschisis is associated with several comorbidities, yet there is much discrepancy in the literature regarding which specific ultrasound markers best predict adverse neonatal outcomes. Future research should include prospective trials with larger sample sizes and use well-defined and consistent definitions of the adverse outcomes investigated with consideration given to IABD.

## 1. Introduction

**Figure 1:**
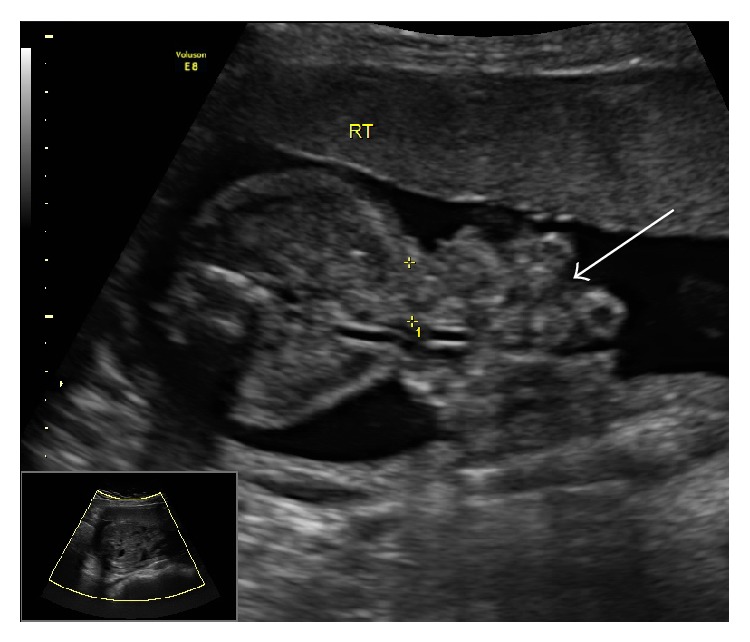
Ultrasound image showing small anterior wall defect beside umbilical cord insertion with small bowel herniation.

Gastroschisis is a congenital abdominal wall defect occurring in approximately 5 in 10,000 live births [1]. As a full thickness defect in the anterior abdominal wall gastroschisis is almost invariably located to the right of the umbilical ring and is characterized by the extrusion of the midgut from the coelom with the absence of a membranous covering (Figure 1) [2]. The pathophysiology of gastroschisis continues to elude clinicians and researchers although risk factors that are consistently associated with the development of this defect include young maternal age, low BMI, race, smoking, low socioeconomic status, recreational drug use, and alcohol consumption during pregnancy [3]. Although the survival rate for infants born with gastroschisis is approximately 90% it is associated with significant morbidity resulting from prolonged hospital stay, delay in time to start oral feeding, time on ventilator, long-term use of total parenteral nutrition (TPN), multiple surgical interventions, and neonatal complications including sepsis, necrotizing enterocolitis, and short bowel syndrome [4, 5].

The condition of the bowel at birth is an important prognostic factor for neonatal comorbidities. Neonates with gastroschisis can be divided into two groups, which have distinct and unique outcomes, based on the presence or absence of associated bowel complications including atresia, necrosis, volvulus, perforation, and ischemia [6]. Optimal management for neonates with gastroschisis is unclear given the controversy in literature regarding which factors most accurately predict neonatal outcomes [7]. As such, this review aims to highlight sonographic predictors of neonatal outcome most commonly reported in the literature including bowel thickness, bowel dilatation, stomach dilatation, stomach herniation, bladder herniation, intrauterine growth restriction (IUGR), abdominal circumference, hyperperistalsis, being small for gestational age (SGA), amniotic fluid index (e.g., polyhydramnios, oligohydramnios, and meconium-stained amniotic fluid), and liver herniation. An improved ability to predict which fetuses are at an increased risk for neonatal complications may assist with appropriate triage, aid in prenatal counseling/medical management of gastroschisis, and encourage multisystem neonatal support to minimize postnatal complications [8, 9].

## 2. Methods

### 2.1. Search Strategy

PubMed and Ovid were queried to identify relevant literature pertaining to antenatal ultrasound predictors of adverse outcome in gastroschisis. To complement the comprehensive Ovid search, a PubMed search was conducted using the search terms “gastroschisis and predictors and outcome” with no filters applied. This yielded 15 papers that were included if they met the following inclusion criteria:

1. antenatal gastroschisis diagnosis,
2. predictors of adverse outcome being the primary focus of study.

All abstracts were reviewed for content relevance and 2 papers were excluded as they were out of scope and focused on the effects of maternal factors and colonic atresia on adverse outcomes. The remaining 13 papers were read in detail and eliminated if the primary outcome of the study was not specifically ultrasound predictors of adverse neonatal outcome; this left four papers for review.

Two additional Ovid searches were conducted to ensure completeness using the Ovid MEDLINE(R) In-Process and Other Non-Indexed Citations and Ovid MEDLINE (R), 1946 to present. The first search used the terms “gastroschisis” and “predictors of outcome.” Each of the terms was searched independently and then combined to generate the new combined search term of “Gastroschisis AND predictors of outcome” which yielded a total of 2 results. One of the two was a duplicate from the previous PubMed search and the other was read and excluded as it failed to satisfy the aforementioned inclusion criteria. The second Ovid search used the key words “gastroschisis” and “ultrasound.” Similar to the first Ovid search, the terms were searched separately and then combined to generate the new search term of “Gastroschisis and Ultrasound” which yielded a total of 91 results. The following limits were then applied:

1. English,
2. humans,
3. publication year: 2009-current.

The publication year limits were set in order to ensure that studies captured were relevant to modern clinical practice. A total of 34 search results remained. All papers were reviewed and 20 were omitted (duplicates or failed to meet inclusion criteria) (see the Appendix). The remaining 12 were then comprehensively reviewed and included. Lastly, reference lists of the included studies were searched for original articles that may meet inclusion criteria. Two papers were retrieved using this method leaving a total of 18 papers included in this review.

### 2.2. Synthesis Strategy

Key details pertaining to our objectives and inclusion criteria were extracted from the 18 papers and tabulated (Table 1). The data extracted included descriptive information including patient characteristics, sample size, study design and analytical methods, prenatal ultrasound markers evaluated, adverse outcomes reported, statistically significant prenatal ultrasound markers predictive of outcome, odds ratios and 95% confidence intervals (OR 95% CI), and *P* values.

## 3. Prenatal Ultrasound Markers and Identification of Adverse Outcomes

### 3.1. Intra-Abdominal Bowel Dilatation (IABD)

**Figure 2:**
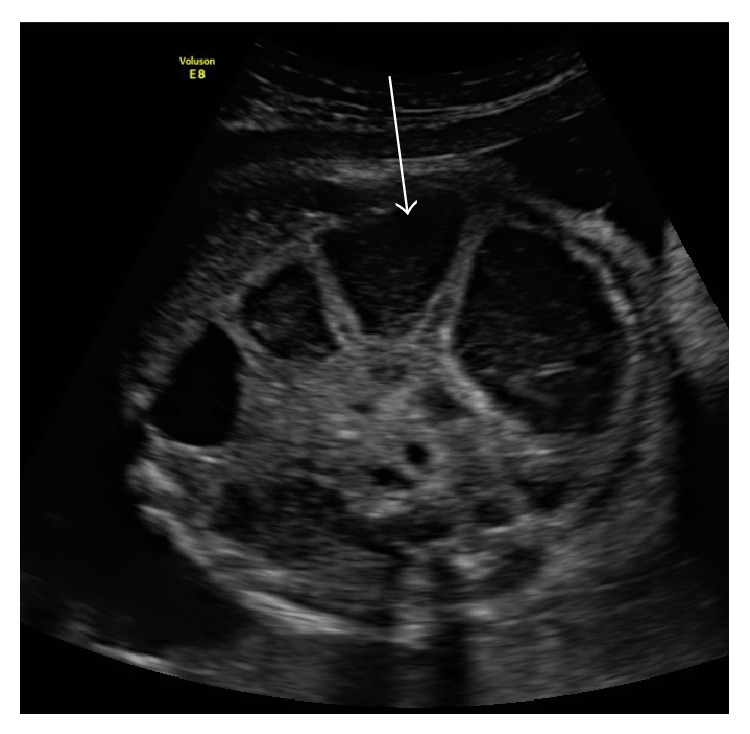
Ultrasound image demonstrating intra-abdominal loops of bowel dilatation in fetal gastroschisis at 33 weeks of gestation.

Several studies report IABD (Figure 2) as a significant predictor of various adverse outcomes in cases presenting with gastroschisis [1, 10–13]. However, heterogeneity among included studies with respect to methodology and diagnostic thresholds for IABD has resulted in contradictory results (Table 1). For instance, Nick et al. [10] completed a single-centre retrospective chart review and report that IABD in the second trimester is a statistically significant predictor of bowel atresia as all infants in this study that presented with IABD were diagnosed with bowel atresia after birth. However, a threshold was not used to identify the presence of IABD and results may vary if only severe dilatation was included. Moreover, IABD in the second trimester was associated with prolonged NICU length of stay (57 days versus 29 days for those without IABD).

Similar results were reported in a single-centre retrospective cohort study by Goetzinger et al. [1] such that patients with IABD >14 mm had 3-fold greater likelihood of bowel atresia (3.1 OR (95% CI: 1.2–8.2)) in comparison to those without IABD (<14 mm). Prolonged stay in the neonatal intensive care unit (NICU) (81 versus 48 days) was also greater for those with IABD. In a single-centre retrospective case-control study by Kuleva et al. [11], it was demonstrated that infants with IABD (>6 mm) were four times more likely compared to those with IABD (<6 mm) to have complex gastroschisis, which they defined as gastroschisis with associated bowel-related complications (e.g., intestinal atresia, perforations, necrosis, and volvulus). In this study subcategorizing cases into “complex” and “simple” gastroschisis aided in predicting morbidity as the infants that were classified as complex gastroschisis required multiple interventions and stoma placement, with a longer time on parenteral nutrition and a prolonged hospital stay [11]. Likewise, Contro et al. [12] reported that infants with IABD (>6 mm) had a fourfold increased risk of presenting with postnatal bowel obstruction. In this same study, IABD was predictive of the need for bowel resections and a second laparotomy, although no odds ratios were reported. Yet, contrary to the other reports, this single-centre retrospective chart review failed to find a significant association between IABD and NICU length of stay [12]. Finally, with respect to adverse outcomes, Houben et al. [13] focused specifically on closing gastroschisis which they defined as the circumferential or partial closure of the ring around the protruding bowel associated with intestinal atresia, bowel ischemia, bowel necrosis, or viable intestine. Similar to the aforementioned studies, this retrospective chart review reports an association between IABD (>10 mm) and closing gastroschisis with associated intestinal atresia. However, these results must be interpreted with caution as they failed to report odds ratios or *P* values.

Only three of the studies reported maternal characteristics, albeit to a limited degree. Furthermore, no statistical tests were completed to determine if there were significant relationships between maternal characteristics and adverse outcome [1, 11, 12]. Maternal prepregnancy weight, body mass index (BMI), and nutrition/lifestyle issues were not reported in any of the five studies and are factors known to influence fetal growth [14, 15]. Collectively, the definition of IABD is inconsistent and diagnostic thresholds varied between studies. Standardized measures are of utmost importance to reliably define a threshold for severe IABD. Only then can consensus be reached in terms of its true clinical significance in predicting adverse outcome. If no or low thresholds are used the prevalence of adverse outcomes is likely overestimated and modifications to current practice may not be warranted. Goetzinger et al. [1] concluded that despite the presence or absence of sonographic findings such as IABD, EABD, and bowel wall thickness, they do not advocate a change in antenatal surveillance or timing of delivery. In contrast, Houben et al. [13] highlighted the importance of early delivery if closing gastroschisis was suspected. In light of these discrepancies it is evident that further research is required in order to reconcile variation in clinical recommendations with respect to timing of delivery and other surgical intervention in fetal gastroschisis.

### 3.2. Intrauterine Growth Restriction (IUGR)

Puligandla et al. [16] define IUGR as insufficient in utero fetal growth based on ultrasound examinations, Doppler flow assessments, and biophysical profiles. Many clinicians support preterm birth for infants with significant IUGR, which is a topic of controversy for infants with gastroschisis [16]. For instance, a retrospective chart review by Nick et al. [10] reviewed several antenatal variables to assess their ability to predict the presence of neonatal bowel atresia. IUGR, defined as birth weight for gestational age of less than the 10th percentile, was found to be a significant predictor as six of ten newborns with atresia presented with IUGR (60%), compared with ten of forty-eight without atresia (21%) [8]. Similarly, in a large retrospective cohort study, Nicholas et al. [17] confirmed a high incidence of IUGR in gastroschisis and an increased risk for adverse neonatal outcomes. In this study, they used a composite definition for adverse neonatal outcome which included neonatal death, prolonged hospital stay, >two surgeries, feeding difficulties, sepsis, and gastrointestinal atresia [17]. Conversely, Puligandla et al. [16] demonstrated that IUGR infants with gastroschisis had equivalent outcomes to infants without IUGR. Furthermore, there was no difference between the two groups regarding the number of surgeries required, days on TPN, days to full enteral feeding (PO), total number of days oral feeding was held (NPO), and the length of hospital stay.

Although several studies have indicated IUGR as a significant predictor [10, 16, 17], others have suggested that the prevalence of IUGR is overestimated up to twofold when compared to the diagnosis of SGA at birth [18]. Reasons for this observation may include the fact that sonographic estimated fetal weight (EFW) calculations heavily rely on abdominal circumference which has been found to be smaller in fetal gastroschisis given that the fetal intestines are protruding through the intestinal wall [18].

Nicholas et al. [17] evaluated a collection of maternal characteristics and lifestyle factors and failed to detect any association with adverse outcome. However, Nick et al. [10] and Puligandla et al. [16] did not report any maternal characteristics or lifestyle factors. Puligandla et al. [16] concluded that, in the context of IUGR, routine premature delivery (<36 weeks) was not advocated. In their retrospective chart review, infants born at less than 37 weeks of gestation had more surgeries, longer time on TPN, longer times to full enteral feeding, and longer lengths of stay, despite excluding those with atresia. Although the findings of this single study are not supportive of elective preterm birth in IUGR the results must be interpreted with caution.

### 3.3. Bowel Wall Thickness

It has been proposed that bowel exposure to amniotic fluid results in progressive bowel injury over time, resulting in a sonographic change in the bowel wall's appearance [1]. Bowel wall thickness as a sonographic predictor for adverse neonatal outcome has been studied less extensively and produced discrepant findings. For instance, Goetzinger et al. [1] suggested prolonged exposure of the fetal bowel to the amniotic fluid results in progressive bowel injury over time and consequently changes the bowel wall appearance. In a retrospective study, Goetzinger et al. [1] demonstrated an increased risk for bowel atresia, necrotizing enterocolitis (NEC), prolonged NICU length of stay, and prolonged time to abdominal wall closure in fetuses with thickened bowel wall greater than 3 mm. Despite reaching statistical significance, it was noted that all cases of thickened bowel wall occurred in fetuses with IABD greater than 14 mm. Thus, it is difficult to interpret what factor independently predicts these outcomes [1]. In contrast, Kuleva et al. [11] examined the difference in prevalence of thickened intestinal wall between cases complicated with simple and complex gastroschisis. Of interest, they reported no significant differences between groups. However, one cannot rule out the possibility that differences may have been observed if a continuous threshold was used for determining the presence or absence of thickened intestinal wall as opposed to simple dichotomous categorization. Similarly, in a separate retrospective cohort study, Janoo et al. [6] did not observe any between-group differences in neonatal outcomes with respect to bowel thickness, although a trend emerged suggesting that an adverse event was more likely with a progressively thicker bowel. Lastly, Davis et al. [9] evaluated the clinical significance of bowel wall thickening of >3 and >4 mm but found no relationship between bowel wall thickness and bowel condition at birth or with poor clinical outcomes.

Although Goetzinger et al. [1] reported maternal characteristics, the study was limited as they only looked at a comparison between fetuses with IABD and those without and bowel wall thickness was not included in the analysis. On the other hand, the remaining three studies failed to comprehensively report maternal characteristics and demographics and did not look at the potential association between these factors and adverse outcomes in fetal gastroschisis [6, 9, 11].

With respect to timing of delivery and bowel thickness, Goetzinger et al. [1] did not support a change in the timing of delivery or antenatal surveillance despite ultrasound findings of EABD, IABD, and bowel wall thickness. Likewise, Janoo et al. [6] found no relationship between gestational age and time to feeding, length of hospital stay, or number of days on ventilator indicating their findings do not suggest elective preterm birth. Despite the agreement between these two studies, properly designed trials are needed prior to making clinical recommendations regarding timing of delivery.

### 3.4. Bowel Dilatation: NOS

Bowel dilatation, not otherwise specified (NOS), refers to the studies that did not differentiate between intra-abdominal bowel dilatation and extra-abdominal bowel dilatation when doing their analyses. For instance, in a retrospective chart review, Garcia et al. [4] reported a significant association between bowel dilatation greater than 25 mm and intestinal abnormalities, lower rate of primary surgical closure, longer periods to achieve full oral feeding, and a prolonged hospital stay. In fact, bowel transverse diameter (BTD) > 25 mm yielded a sensitivity of 38%, a specificity of 87%, a positive predictive value (PPV) of 38%, and a negative predictive value (NPV) of 87%. Similar results were observed in another retrospective chart review by Long et al. [19] who reported that bowel dilatation greater than 20 mm was predictive of a higher infant mortality rate and a prolonged time on parenteral nutrition (PN). Although infants with bowel dilatation spent an average longer time on PN the median number of days between the two groups was not different [19]. In a secondary analysis evaluating the effect of atresia on the number of days spent on PN, independent of bowel dilatation, a significant difference was found between those without atresia (median 20 days) versus those with atresia (median 65 days) [19]. These findings illustrate the significant effect that adverse outcomes, such as bowel atresia, can have on secondary neonatal outcomes. In contrast to the studies by Garcia et al. [4] and Long et al. [19], the retrospective chart review done by Wilson et al. [5] reported no significant association between bowel dilatation (IABD, EABD, or both) greater than 20 mm and adverse outcome. This discrepancy may be due to smaller sizes and inadequate power to detect change (e.g., *n* = 87 cases [5] versus *n* = 170 [19] and *n* = 94 [4] cases). Similarly, Davis et al. [9] failed to find a significant relationship between bowel dilation and adverse neonatal outcomes and should be carefully interpreted as the lack of availability of ultrasound records (*n* = 25) may have attenuated a potential relationship. Nonetheless, despite the clear association between bowel dilatation and adverse outcome, Garcia et al. [4] do not recommend elective preterm delivery as it may add further hazard to the inherent surgical morbidity inherently present and that prolonging delivery beyond 37 weeks of gestation does not serve any benefits.

### 3.5. Liver Herniation

Although bowel herniation is routinely observed in fetal gastroschisis, liver herniation is less common [20]. As a result, recent literature often categorizes infants into “complex” and “simple” gastroschisis but the presence of liver herniation is not specifically evaluated [20]. In a retrospective chart review, McClellan et al. [20] aimed to evaluate the prognosis of liver herniation in gastroschisis and found that it was significantly associated with a higher rate of mortality. The survival rates were 43% and 97% for gastroschisis with liver herniation and without, respectively [20]. The extent of liver herniation appeared to predictive of comorbidities, including pulmonary hypoplasia, and poor outcome. Of the 7 patients with herniated liver, 3 only had a small portion of the liver herniated and did not seem to be affected [20]. In contrast, the remaining 4 had a larger portion of the liver herniated, had a mortality rate of 100%, and were more likely to require large silos for closure [20]. Despite the apparent association between liver herniation and adverse neonatal outcome, there is limited research on this topic (1 study, *n* = 117); therefore in order to draw a firm conclusion in regard to clinical recommendations, further research must be conducted.

### 3.6. Bladder Herniation

Similar to liver herniation, bladder herniation is observed less frequently in gastroschisis patients, with an incidence varying from 4.3% to 14% [21]. Fetuses with gastroschisis have a greater risk of stillbirth during the third trimester and fetal distress which is likely partially related to cord compression due to the herniated bowel. In a retrospective cohort study, Mousty et al. [21] hypothesized that bladder evisceration could cause the cord to be more prone to compression thus increasing perinatal mortality and fetal distress. Mousty et al. [21] was the first study to evaluate the specific outcome (e.g., intrauterine fetal demise (IUD) and neonatal death) of infants with bladder herniation. Of the six infants, the indications for delivery included one IUD, three fetal distresses (i.e., abnormal home fetal heart monitoring), one ultrasound abnormality (i.e., bowel hyperechogenicity and pyelectasis), and one planned C-section. These results appear to support a relationship between bladder herniation and adverse outcome. Therefore in cases such as these, increased surveillance may be justified. However, future study is required as current investigations fail to report odds ratios or *P* values.

### 3.7. Delta Dilatation and Final Bowel Dilatation

In a retrospective chart review, Janoo et al. [6] defined delta dilation as final bowel dilatation minus baseline bowel dilatation, which was taken from the first ultrasound readings. This review reported no differences in adverse neonatal outcomes with regard to bowel dilatation and bowel thickening, although there was a significant association between delta dilatation (at 4 mm) and final dilatation to time to feeding. However, given the limited research with small sample sizes on this topic (1 study, *n* = 19) these results must be interpreted with caution with respect to their direct clinical impact.

## 4. Prenatal Ultrasound Markers Likely Unrelated to Adverse Outcome

### 4.1. Abdominal Circumference (AC)

AC measures are smaller in infants with gastroschisis in part because the intestines protrude through the abdominal wall defect [18]. Consequently, this then leads to a false positive appearance of IUGR which in turn leads to unnecessary interventions (i.e., elective preterm delivery) [18]. For example, Ajayi et al. [18] using a retrospective chart review examined AC less than the 5th percentile and its effect on several adverse outcomes including, mortality, primary closure, necrotizing enterocolitis, short gut syndrome, length of stay, days intubated, days until room air oxygen, days until full enteral feeding, and days on TPN. Neonatal outcomes in patients with small AC (<5th percentile) were similar to those with a normal AC. Similarly, Payne et al. [22] validated previous findings that AC less than 5th percentile had no predictive value for either gastrointestinal complications or the need for a silo. The concordant results of Ajayi et al. [18] and Payne et al. [22] may be attributed to similar designs (i.e., both retrospective chart reviews) or attention to confounding variables. Payne et al. [22] accounted for various maternal demographics and clinical characteristics including age, race, marital status, and cigarette use and examined their relationship with hospital length of stay. However, none of the relationships appeared statistically significant. Despite their similar study designs, Ajayi et al. [18] did not examine any maternal parameters. Overall, the concordance between the three studies reporting these outcomes suggests that abdominal circumference <5th percentile is of little concern to clinicians for infants with gastroschisis as there have been no significant relationships found between AC <5th percentile and adverse neonatal outcome of any kind.

### 4.2. Stomach Herniation and Dilatation

In a retrospective cohort study, Nicholas et al. [17] revealed a slightly higher incidence of adverse outcome in fetuses with stomach dilatation, but the data failed to reach statistical significance. Similarly, Kuleva et al. [11] compared the prevalence of stomach herniation and dilatation between the simple gastroschisis group and the complex gastroschisis group. In agreement with the previous findings, this retrospective case control study confirmed that there was no significant difference in prevalence between the two groups suggesting that stomach herniation and dilatation were not predictive markers. Lastly, using a retrospective chart review, Alfaraj et al. [23] reported comparable results with regard to stomach dilatation. Yet, gastric dilatation was not predictive of the presence of neonatal bowel atresia, necrosis, or perforation. There were also no statistically significant differences in the need for intestinal resection, age at full enteral feeding, length of hospital stay, presence of short bowel syndrome, or neonatal death. However, in contrast to the above studies, Ajayi et al. [18] revealed that meconium-stained amniotic fluid at delivery was more common in fetuses presenting with gastric dilatation (53%) than in those without (24%) (*P* = 0.017). Both Kuleva et al. [11] and Alfaraj et al. [23] report few, if any, maternal characteristics, demographics, or lifestyle factors. On the contrary, Nicholas et al. [17] evaluation several maternal and lifestyle factors and assessed their association with adverse outcome but results yielded no significance relationships. Overall, a significant association between stomach herniation or dilatation and adverse neonatal outcome remains to be conclusively demonstrated.

### 4.3. Extra-Abdominal Bowel Dilatation (EABD)

**Figure 3:**
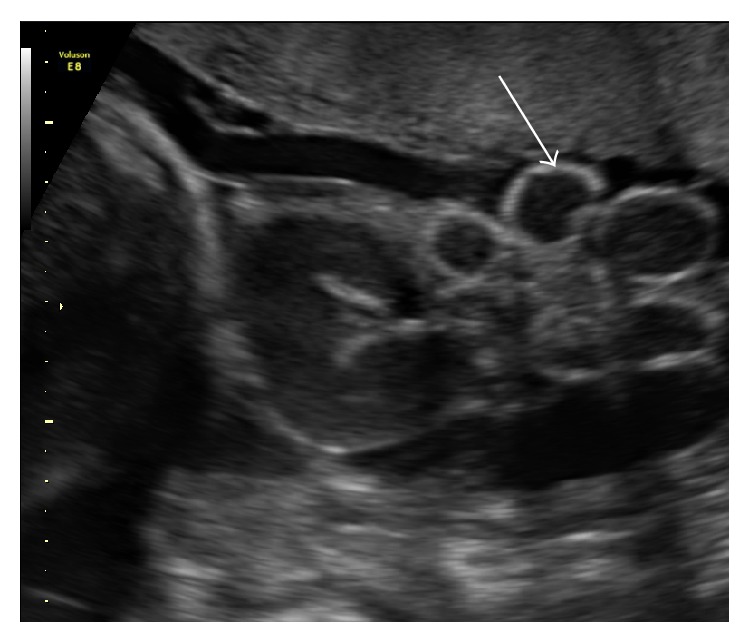
Ultrasound image showing loops of bowel floating free in amniotic fluid in a fetus at 31 weeks of gestation.

**Figure 4:**
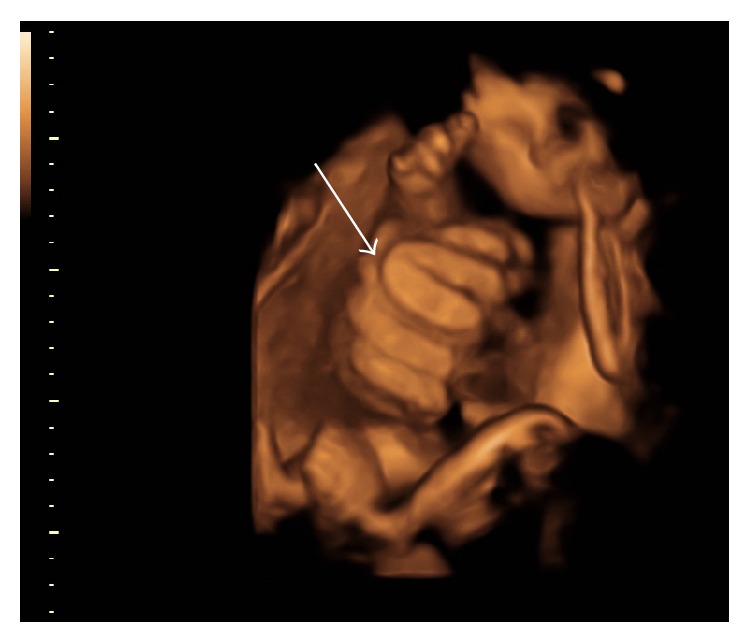
3D ultrasound image demonstrating free loops of intestine in amniotic cavity at 34 weeks.

Dilation of the herniated portion of the fetal bowel may be more reflective of impaired peristalsis rather than true obstruction [1]. This hypothesis appeared to be consistent with the findings of the four studies evaluated [1, 11, 12, 24]. Extra-abdominal bowel dilatation (Figures 3 and 4) was common in many of the studies included in this review despite no association between EABD of any threshold and adverse outcome [1, 11, 12, 24]. For instance, in a retrospective chart review, Contro et al. [12] frequently observed EABD >6 mm but failed to find an association with adverse outcomes. Similarly, both Kuleva et al. [11] and Goetzinger et al. [1] used EABD >6 mm as a threshold and noted that it was not predictive of complex gastroschisis or bowel atresia, respectively. With respect to study design, Kuleva et al. [11] and Goetzinger et al. [1] were retrospective case-control and retrospective cohort studies, respectively. Lastly, using a retrospective chart review, Mears et al. [24] did not find EABD >10 mm to be predictive of adverse postnatal outcomes and in fact noted that the group with EABD (versus IABD, both, or none) were more likely to have primary closure. However, this association may spuriously have been falsely detected due to the smaller sample size (i.e., *n* = 47). Of the maternal factors reported, all four studies mention solely maternal age, with the exception of Kuleva et al. [11] whom also reports parity. None of the studies tested for a statistically significant relationship between maternal characteristics, lifestyle factors, and demographics and adverse outcome. Overall, the current data suggest that EABD of any threshold is not predictive of adverse neonatal outcome and may serve as an ultrasound marker to guide clinical recommendations concerning antenatal surveillance and timing of delivery.

### 4.4. Future Directions

There is much discrepancy in the literature regarding significant predictors of adverse outcome in fetal gastroschisis. All of the studies included in this review were retrospective in nature (i.e., chart reviews, cohort, or case-control studies). Moving forward, in order to help eliminate bias and discordant findings, prospective studies examining antenatal sonographic markers and their potential associations with adverse neonatal outcomes should be conducted. Although it is difficult to get a large number of patients with fetal gastroschisis given the low prevalence of the condition it is essential to compile a larger patient database to eliminate incongruity, maximize power, and produce valid and reliable results. Furthermore, establishing a consistent definition of adverse outcome (e.g., complex gastroschisis, death, prolonged NICU length of stay, and multiple surgical interventions) with specific attention given to the most sensitive thresholds for bowel dilatation and bowel wall thickness is encouraged. In the current review all published studies used inconsistent definitions of bowel dilatation and bowel wall thickness; therefore the discrepant results are expected. It was noted by Garcia et al. [4] that their data confirmed previous reports of a significant and positive correlation between bowel diameter and gestational age. This emphasizes the importance of adjusting the definition of bowel dilatation with varying gestational age, a covariate which should be accounted for in future investigations to improve accuracy.

## 5. Conclusion

Fetal gastroschisis is associated with several morbidities that may lead to secondary adverse outcomes including prolonged time to start oral feeding, time on ventilator, long-term use of TPN, multiple surgical interventions, and neonatal complications including sepsis, necrotizing enterocolitis, and short bowel syndrome [4, 5]. Despite this, there is still much discrepancy in the literature regarding which ultrasound predictors are most sensitive and clinically relevant to the prediction of adverse neonatal outcomes. Future prospective studies with adequate power and appropriate samples sizes that employ standardized definitions of adverse outcome will help generate reliable and valid data that can be used to inform patient care and ultimately improve maternal-fetal health.

## Figures and Tables

**Table 1:** Summary of prenatal ultrasound markers predictive of adverse outcome in the included studies.

Study	Study characteristics	Sample size [*n*]	Methods	Prenatal UM evaluated	Adverse outcome	Prenatal UM predictive of outcome	OR (95% CI)	*P* value
Puligandla et al., 2004 [16]	Retrospective analysis. Infants born with GS between 1990 and 2000	113	Analysis of variance (ANOVA), Student's *t*-test, and Fisher's exact tests and linear and logistic regression used for statistical analysis; *P* < 0.05 = significant	IUGR	Number of surgeries Days on TPN Days to full PO Days NPO Length of stay (days)	None	NR	NS

Nick et al., 2006 [10]	Retrospective review from January 1998 to August 2004. All neonates delivered with GS and admitted to Vanderbilt University Medical Centre	72	Binary variables analyzed with Fisher's exact test; continuous variables analyzed by logistic regression; Wilcoxon's rank sum test determined if the number of days to complete closure and LOS was different between neonates with and without atresia; *P* < 0.05 = significant	IABD (no threshold) Oligohydramnios IUGR <10th percentile Abnormal umbilical artery Doppler velocimetry	Small-bowel atresia LOS in NICU LOS in hospital Time to complete closure Discharge status of infant	IABD IUGR IABD None	NR	<0.0001 0.0199 0.0052 NS NS NS

Davis et al., 2009 [9]	Retrospective analysis of neonates with GS at a single institution between June 1998 and March 2007	46	Comparisons made using Fisher's exact test, Pearson's test, Student's *t*-test for continuous variables, or ANOVA; *P* < 0.05 = significant	Bowel dilatation (>10, >17, >20 mm) Bowel wall thickness (>3, >4 mm) AFI	Bowel atresia Necrotic bowel Bowel stenosis In utero volvulus ^∗∗^Other outcomes explored but all NS	None	NR	NS

Houben et al., 2009 [13]	Retrospective review of all infants born with GS at King's College Hospital (UK) from August 1994 to December 2007	46	Data quoted as median (range)	IABD > 10 mm Growth restrictions Hyperperistalsis	Closing gastroschisis (defined as circumferential or partial closure of the ring around protruding bowel associated with intestinal atresia, bowel ischemia, bowel necrosis, or viable intestine)	IABD	NR	NR

Payne et al., 2009 [22]	Retrospective analysis of all GS patients born between January 1990 and December 2007 admitted at NICU of the Children's Hospitals and Clinics of Minnesota, Minneapolis Campus	155	Normality of data examined using Shapiro-Wilk test; nonnormal distributed variables were summarized as median and range; univariate analyses performed using Wilcoxon's rank-sum or Fisher's exact tests; linear regression used to determine association between parenteral nutrition and LOS; variables associated with LOS at *P* < 0.10 were included in multiple regression; *P* < 0.05 = significant	AFI <5th percentile AC <5th percentile Dilated intestine (>10 mm, >18 mm)	GI complication Requiring a silo Primary repair	Dilated intestine >10 mm Dilated intestine >18 mm None	NR	0.01 0.003 NS NS

Nicholas et al., 2009 [17]	Retrospective cohort study at Washington University Medical Center from 1991 to 2006	80	Univariable and multivariable statistical analysis; backward stepwise logistic regression used to identify variables in final prediction model; *P* < 0.10 = significant in univariate analysis; predictive effectiveness of final model evaluated using area under receiver operating characteristic curve (AUC-ROC)	Dilated bowel >10 mm Dilated stomach IUGR Hyperperistalsis AFI anomalies	Composite: death, prolonged hospital stay, >2 surgeries, feeding difficulties, sepsis, atresia	IUGR	2.7 (1.0–7.3)	0.05

Ajayi et al., 2011 [18]	Retrospective review of pregnancies complicated by GS between 2000 and 2008	74	Categorical data analyzed with Fisher's exact test; statistical normality evaluated using Shapiro-Wilk statistic; continuous variables that were normally distributed compared using Student's *t*-test and continuous variables not normally distributed compared using Wilcoxon rank sum; *P* < 0.05 = significant	AC <2.5th percentile	Mortality Primary closure Necrotizing enterocolitis Short gut syndrome LOS Days intubated Days until room air oxygen Days until full enteral feeding Days on TPN ^∗∗^Other outcomes examined but all found to be NS	None	NR	NS

Alfaraj et al., 2011 [23]	Retrospective study of singleton neonates with GS delivered at Mount Sinai Hospital with postnatal care at the Hospital for Sick Kids in Toronto, Canada, from January 2001 to February 2010	98	Chi-square or Fisher's exact test used for categorical data; continuous variables presented as mean ± SD; continuous variables compared used Student's *t*-test or Mann-Whitney *U* test; conventional *P* values corrected for multiple comparisons using Bonferroni method; *P* < 0.05 = significant	Gastric dilatation >2 SD above normal value SGA <5th percentile Polyhydramnios (>25 cm)	Meconium stained amniotic fluid Intestinal atresia, necrosis, or perforation Need for intestinal resection Age at full enteral feeding (days) LOS (days) Short bowel syndrome Neonatal death ^∗∗^Other outcomes noted at but NS	Gastric dilatation None	NR	0.017 NS NS NS NS NS NS

Contro et al., 2010 [12]	Retrospective study of all GS cases between November 1998 and September 2008	48	Categorical data compared with Fisher's exact test; normality of continuous data tested using Kolmogorov-Smirnoff test; comparisons carried out using Student's *t*-test or Mann-Whitney *U* test; *P* < 0.05 = significant	IABD > 6 mm EABD > 6 mm	Bowel obstruction Bowel resection Second laparotomy Time in NICU (days) ^∗∗^Other outcomes looked at but no *P* value reported as they were NS	IABD None	4.05 (1.12–14.7) NR	0.037 0.045 0.021 0.062
Garcia et al., 2010 [4]	Retrospective study of singletons with a prenatal diagnosis of GS at a tertiary center for fetal medicine in Brazil from January 1997 to August 2009	94	Cut-off value for prediction determined in ROC curve; cases grouped according to bowel dilatation and compared with chi-square and Fisher's exact test and Mann-Whitney *U* test	Bowel dilatation >25 mm	Intrauterine fetal death (IUD) Neonatal death (NND) Volvulus Perforation Any bowel complications Atresia Necrosis Time to oral feeding (days) LOS (days)	None Bowel dilatation	NR	NS NS NS NS 0.003 0.007 0.03 0.02 0.04

Mears et al., 2010 [24]	Retrospective study of all cases of isolated GS diagnosed antenatally from 2004 to 2008	47	Spearman correlations used to explore relationships between antenatal findings and outcome measurements. Differences between groups examined with Kruskal-Wallis and Mann-Whitney *U* tests; *P*-value <0.05 = significant	IABD > 10 mm EABD > 10 mm Both IABD and EABD	Type of surgical repair (primary, silo, patch, or stoma) Days on TPN Complications Death	EABD predicted primary closure None	NR	0.03 NS NS NS

Kuleva et al., 2012 [11]	Retrospective case-control study of all antenatal diagnoses of isolated GS from 1999 to 2010	105	Normality of continuous data tested using Kolmogorov-Smirnoff test; between-group comparisons using Fisher's exact test, Mann-Whitney *U* test, or Student's *t*-test; relationship between prenatal ultrasound markers, complex GS and adverse outcome tested by chi-square test and logistic univariate and multivariate regression; all *P* values <0.05 = significant	Thickened intestinal wall IABD > 6 mm EABD > 6 mm Dilated stomach Stomach herniation SGA	CGS IUFD ND	IABD None	4.13 (1.32–12.90)	0.018 NS NS

Long et al., 2011 [19]	Cases of antenatally diagnosed GS were identified from an in-house database of antenatal ultrasound scans performed in the Fetal Management Unit at St Mary's Hospital, Manchester, from January 1998 to December 2007	170	Chi-square test used to compare categorical outcomes and Fisher exact test used where numbers of included individuals were <10; Mann-Whitney *U* test used for nonparametric data; *P* value <0.05 = significant	Bowel dilatation >20 mm	GA at delivery Days on PN Death Surgery for IF BW at delivery Intestinal atresia	Bowel dilatation None	NR	0.02 0.03 0.01 NS NS 0.07

McClellan et al., 2011 [20]	Retrospective review of patients undergoing surgery for GS at the University of California Los Angeles Medical Center from 1995 to 2010	117	Logistic regression used to compare association between mortality of gastroschisis patients with liver herniation with those without	Liver herniation	Mortality	Liver herniation	NR	*P* < 0.001

Mousty et al., 2012 [21]	Retrospective cohort study of six singletons with GS associated with secondary fetal bladder herniation managed at a tertiary referral center between 2001 and 2010	6	No statistics presented	Bladder herniation	Mortality	Bladder herniation	NR	

Wilson et al., 2012 [5]	Retrospective review of all cases of GS evaluated prenatally at the Center for Advanced Maternal Fetal Care, September 2007–June 2010	89	Categorical data compared with chi-square test, Student's *t*-test; *P* < 0.05 = significant; linear regression used to estimate association between days in NICU and presence of any bowel dilatation	Bowel dilatation (IABD, EABD, or both) >10 mm	Gestational age at birth Birth weight at delivery Length of NICU admission Number of surgeries	None	NR	NS

Janoo et al., 2013 [6]	Retrospective cohort study, all cases of GS managed at West Virginia University Hospital Morgantown 1998–2002	19	*P* value below 0.05 = significant	Bowel thickness Final bowel dilatation Delta dilatation	Time to feeding Number of days on ventilator Number of days in hospital	Final bowel dilatation Delta dilatation None	NR	0.023 0.007 NS NS

Goetzinger et al., 2014 [1]	Retrospective cohort study, patients carrying singletons diagnosed with GS, at Washington University Medical Center Division of Ultrasound and Genetics from 2001 to 2010	94	Normality tested using Kolmogorov-Smirnov test; Student's *t*-tests and Mann-Whitney *U* tests used to compare continuous variables; chi-square and Fisher's exact tests used to compare dichotomous categorical variables	IABD (<6, >10, >14, and >18 mm) EABD Bowel-wall thickening (>3 mm)	Bowel atresia NICU length of stay (days) Bowel atresia NE NICU length of stay (days) Time to abdominal wall closure (days)	IABD > 14 mm Thickened bowel wall	3.1 (1.2–8.2)	0.01 0.02 0.04 0.03 0.03 0.02

GS: gastroschisis; IUGR: intrauterine growth restriction; TPN: total parenteral nutrition; PO: time to full enteral feedings; NPO: total number of days feeding was held; IABD: intra-abdominal bowel dilatation; NICU: neonatal intensive care unit; AFI: amniotic fluid index; AC: abdominal circumference; GI: gastrointestinal; LOS: length of stay; SGA: small for gestational age; EABD: extra-abdominal bowel dilatation; CGS: complex gastroschisis; IUFD: intrauterine fetal demise; ND: neonatal death; GA: gestational age; IF: intestinal failure; BW: birth weight; NE: necrotizing enterocolitis.

^∗∗^Denotes explanation that follows.

**Table 2:** Table of excluded studies.

Study excluded	Reason for exclusion
J. Boutros, M. Regier, E.D. Skarsgard, “Is timing everything? The influence of gestational age, birth weight, route, and intent of delivery on outcome in gastroschisis,” *Journal of Pediatric Surgery,* vol. 44, pp. 912–917, 2009.	Alternate study focus Focus: GA, BW, route, intent of delivery, timing of delivery

B.T. Bucher, I.G. Mazotas, B.W. Warner et al., “Effect of time to surgical evaluation on the outcomes of infants with gastroschisis,” *Journal of Pediatric Surgery*, vol. 47, pp. 1105–1110, 2012.	Alternate study focus Focus: effect of time to surgery on gastroschisis outcome

K.N. Cowan, P.S. Puligandla, J.M, Laberge et al., “The gastroschisis prognostic score: reliable outcome prediction in gastroschisis,” *Journal of Pediatric Surgery,* vol. 47, pp. 1111–1117, 2012.	Alternate study focus Focus: bowel appearance after birth

O. Ergun, E. Barksdale, F.S. Ergun et al., “Timing of delivery of infants with gastroschisis influences outcome,” *Journal of Pediatric Surgery,* vol. 40, pp. 424–428, 2005.	Alternative study focus Focus: timing of delivery

D.G. Farmer, R.S. Venick, J. Colangelo et al., “Pretranslplant predictors of survival after intestinal transplantation: analysis of a single-centre experience of more than 100 transplants,” *Transplant journal, *vol. 90, no. 12, pp. 1574–1580, 2010.	Alternative study focus Focus: intestinal transplants

C.L. Snyder, “Outcome analysis for gastroschisis,” *Journal of Pediatric Surgery,* vol. 34, no. 8, pp. 1253–1256, 1999.	Date of publication too old (wanted to stay relevant with research and practice)

C.W. Synder, J.R. Biggio, P. Brinson et al., “Effects of multidisciplinary prenatal care and delivery mode on gastroschisis outcomes,” *Journal of Pediatric Surgery*, vol. 46, pp. 86–89, 2011.	Alternative study focus Focus: multidisciplinary prenatal care and mode of delivery

J.A. Mills, Y. Lin, Y.C. MacNab et al., “Perinatal predictors of outcome in gastroschisis” *Journal of Perinatology*, vol. 30, pp. 809–813, 2010.	Alternative study focus Focus: SNAP-II score

H.F. Tsai, Y.C. Cheng, H.C. Ko et al., “Prenatal diagnosis of fetal gastroschisis using three-dimensional ultrasound: Comparison between 20th and 21st centuries,” *Taiwanese Journal of Obstetrics and Gynecology*, vol. 52, pp. 192–196, 2013.	Alternative study focus Focus: comparison of diagnosis using 3D ultrasound between 20th and 21st centuries

D. Baud, A. Lausman, M.A. Alfaraj et al., “Expectant management compared with elective delivery at 37 weeks for gastroschisis,” *American College of Obstetricians and Gynecologists*, Vol. 121, no. 5, pp. 990–998, 2013.	Alternative study focus Focus: timing of delivery

S. Emil, N. Canvasser, T. Chen et al., “Contemporary 2-year outcomes of complex gastroschisis,” *Journal of Pediatric Surgery,* vol. 47, pp. 1521–1528, 2012.	Alternative study focus Focus: complex versus simple gastroschisis

T. Kumar, R. Vaughan, and M. Polak, “A proposed classification for the spectrum of vanishing gastroschisis,” *European Journal of Pediatric Surgery*, vol. 23, pp. 72–75, 2013.	Alternative study focus Focus: classifying vanishing gastroschisis

E.R. Christison-Lagay, C.M. Kelleher, and J.C. Langer, “Neonatal abdominal wall defects,” *Seminars in Fetal *&* Neonatal Medicine*, vol. 16, pp. 164–172, 2011.	Alternative study focus Focus: diagnosis and surgical management

P. Chaudhury, S. Haeri, A.L. Horton et al., “Ultrasound prediction of birthweight and growth restriction in fetal gastroschisis,” *American Journal of Obstetrics *&* Gynecology*, vol. 203, pp. 395 (e1–5), 2010.	Alternative study focus Focus: EFW calculations

J.H. Chung, C. Norton, and S. Emil, “Ultrasound abnormalities spurred delivery and neonatal surgery,” *American Journal of Obstetrics *&* Gynecology*, vol. 201, pp. 332 (e1-2), 2009.	Case study

L.O. Abdur-Rahman, N.A. Abdulrasheed, and J.O. Adeniran, “Challenges and outcomes of management of anterior abdominal wall defects in a Nigerian tertiary hospital,” *African Journal of Paediatric Surgery*, Vol. 8, no. 2, pp. 159–163, 2011.	Alternative study focus Focus: challenges and outcomes of management of abdominal wall defects

A.J.A. Holland, K. Walker, and N. Badawi, “Gastroschisis: an update,” *Pediatric Surgery International*, vol. 26, pp. 871–878, 2010.	Alternative study focus Focus: diagnosis, treatment, risk factors, neurodevelopmental outcomes, incidence

G. Tonni, P. Pattaccini, A. Ventura et al., “The role of ultrasound and antental single-shot fast spin-echo MRI in the evaluation of herniated bowel in case of first trimester ultrasound diagnosis of fetal gastroschisis, “*Archives of Gynecology and Obstetrics*, vol. 283, pp. 903–908, 2011.	Case study

M.E. Brindle, H. Flageole, and P.W. Wales, “Influence of maternal factors on health outcomes in gastroschisis: a Canadian population-based study,” *Canadian Pediatric Surgery Network*, vol. 102, no. 1, pp. 45–52, 2012.	Alternative study focus Focus: maternal (nonsonographic) factors

I. Karnak, A.O. Ciftci, M.E. Senocak et al., “Colonic atresia: surgical management and outcome,” *Pediatric Surgery International*, vol. 17, no. 8, pp. 631–635, 2001.	Date of publication too old (wanted to stay relevant with research and practice)

S. Paranjothy, H. Broughton, A. Evans et al., “The role of maternal nutrition in the aetiology of gastroschisis: an incident case-control study,”* International Journal of Epidemiology, vol. *41, no. 4, pp. 1141–1152, 2012.	Alternative study focus Focus: maternal nutrition

S. Uludag, O. Guralp, M. Akbas et al., “Bladder extrophy,” *Fetal & Pediatric Pathology*, vol. 31, no. 4, pp. 225–229, 2012.	Alternative study focus Focus: bladder exstrophy

K. Ono, A. Kikuchi, K.M. Takikawa et al., “Hernia of the umbilical cord and associated ileal prolapse through a patent omphalomesenteric duct: prenatal ultrasound and MRI findings,” *Fetal Diagnosis *&* Therapy*, vol. 25, no. 1, pp. 72–75, 2009.	Alternative study focus Focus: hernia of umbilical cord and ileal prolapse

I. Juhasz-Boss, R. Goelz, E.F. Solomayer et al., “Fetal and neonatal outcome in patients with anterior abdominal wall defects (gastroschisis and omphalocele),” *Journal of Perinatal Medicine*, vol. 40, no. 1, pp. 85–90, 2012.	Alternative study focus Focus: comparison of outcomes between gastroschisis and omphalocele

M Kuleva, L.J. Salomon, G. Benoist et al., “The value of daily fetal heart rate home monitoring in addition to serial ultrasound examinations in pregnancies complicated by fetal gastroschisis,” *Prenatal Diagnosis*, Vol. 32, no. 8, pp. 789–796, 2012.	Alternative study focus Focus: benefit of daily fetal heart rate home monitoring and serial ultrasound examinations

A.M. Kassa, and H.E. Lilja, “Predictors of postnatal outcome in neonates with gastroschisis,” *Journal of Pediatric Surgery*, vol. 46, no. 11, pp. 2108–2114, 2011.	Alternative study focus Focus: nonultrasound predictors of secondary outcome

N.H. Grant, J. Dorling, and J.G. Thornton, “Elective preterm birth for fetal gastroschisis,” *Cochrane Database of Systematic Reviews*, 2013.	Alternative study focus Focus: timing of delivery

## Appendix

See Table 2.
